# Computer-Assisted Learning Applications in Health Educational Informatics: A Review

**DOI:** 10.7759/cureus.1559

**Published:** 2017-08-10

**Authors:** Faiq Shaikh, Faisal Inayat, Omer Awan, Marlise D Santos, Adnan M Choudhry, Abdul Waheed, Dilkash Kajal, Sagun Tuli

**Affiliations:** 1 Institute of Computational Health Sciences, University of California San Francisco; 2 Department of Medicine, New York-Presbyterian Hospital, Weill Cornell Medical College, New York City, NY, USA; 3 Department of Radiology, Temple University Hospital; 4 Department of Radiology, University of Ottawa, Ottawa, on; 5 Neuroscience Research & Medical Education Program, IMCHF, Montreal, Qc; 6 Department of Family Medicine, Milton S. Hershey Medical Center, Penn State College of Medicine, Hershey, Pa; 7 Department of Radiology, University of Toronto, Toronto, on; 8 Department of Neurosurgery, Metro-West Medical Center, Boston, Ma

**Keywords:** health education, informatics, computer-assisted learning

## Abstract

Computer-assisted learning (CAL) as a health informatics application is a useful tool for medical students in the era of expansive knowledge bases and the increasing need for and the consumption of automated and interactive systems. As the scope and breadth of medical knowledge expand, the need for additional learning outside of lecture hours is becoming increasingly important. CAL can be an impactful adjunct to conventional methods that currently exist in the halls of learning. There is an increasing body of literature that suggests that CAL should be a commonplace and the recommended method of learning for medical students. Factors such as technical issues that hinder the performance of CAL are also evaluated. We conclude by encouraging the use of CAL by medical students as a highly beneficial method of learning that complements and enhances lectures and provides intuitive, interactive modulation of a self-paced curriculum based on the individual’s academic abilities.

## Introduction and background

The vast changes seen in the world of information sciences have had a huge impact on the access, delivery, and academics of healthcare. The surge in medical information and improving methods in diagnostics and therapeutics suggest that healthcare professionals have the challenge of keeping up with an ever-increasing scope of medical knowledge. Treatment options are becoming more complex, resulting in an increase in sophisticated skill set requirements, which are also becoming increasingly technology oriented. Keeping up to date in this field is necessary if one is to maintain clinical proficiency and function along with due diligence as a professional healthcare worker.

## Review

What Is Health Informatics?

Health informatics may be defined as a field of information science that encompasses tools, expertise, and the understanding needed to allow information to be managed, collected, used, and shared for supporting the performance of healthcare. The role of healthcare is to aid in the prevention, diagnosis, treatment, and management of illnesses; informatics is related to a systematic approach to processes and the utilization of information in all of these aspects of healthcare. Healthcare organizations have the responsibility to promote good health and well-being through their vast facilities and services and health informatics facilitates that process throughout the spectrum [1].

Uses of and Need for the Application

Time is a big constraint in learning in the current age. With the ever-expanding scope of medical knowledge, it is evident that there is a growing need for a more systematic and organized approach to acquire, process, and assimilate information. The need for such a productivity tool is further pronounced when taking into account varying scholastic performances among individuals. Especially as a medical student, substantial learning and revisions are required outside of lecture hours, which includes audiovisual aids, microscopic and macroscopic specimens, structural models and mannequins, and procedural training. Computer-assisted learning (CAL) allows for asynchronous learning or learning that can be most optimal for a learner at his/her pace when he/she deems as the best time to learn. The effective incorporation of these resources into the study plan requires the use of novel approaches using information systems, software development, and imaginative techniques to create high-yield simulation modules and e-learning tools. The use of the methods would lead to a better understanding of core concepts and adherence to techniques and protocols, resulting in overall higher clinical proficiency in successfully trained doctors.

Proposed Informatics Application

CAL is defined as the use of computers to support the learning and training of individuals [2]. One may argue that a common way to use computers effectively is by the application of software created for different academic subjects. Programs have been designed and created to be enormously helpful in educating people and assisting them by virtue of improved time management, higher-fidelity simulation modules, and more intuitive feedback systems. The ideal aim would be for the computers to become another "tutor," "peer," and ‘‘examiner" in order to provide a holistic insight into the educational process.

CAL systems include interactive assessment modules and informative content and provide essential feedback to advise on the progress of students. It has been postulated that CAL consists of a range of computer-based packages that focus on providing interactive instruction in a specific subject area [3]. Much of the literature review has observed its effectiveness in supporting the learning of the curriculum for medical students.

Advantages of CAL – Learning Pace and Comprehension

In general, CAL in medical education has demonstrated successful results in the attainment of learning outcomes [4]. Given the fast pace of advancement in medical education, the application of interactive educational software programs allows for a more self-directed and self-paced learning experience, also known as asynchronous learning [5]. There are a variety of multimedia resources offering a personalized learning experience tailored to meet the needs of individual students and offering them essential feedback. Due to the varying learning styles of people, it is advisable that students have enough time to consolidate their learning and comprehend it fully. CAL modules can be applied to visual, audio, and kinesthetic learners. Furthermore, as opposed to just learning in lectures and training sessions, which are bounded by time slots, students can learn at their own time and convenience [6]. In support of this are Haq and Dacre, who believe individuals work at their own pace and educational modules like this can prove to be key in their curriculum [7].

In support of how CAL assists students with their differing learning abilities, Jaffe and Lynch argue that students with varying abilities/levels of training have the opportunity, through CAL, to follow their individual instruction route that enables them to absorb the same comprehensive material [8]. Coiera states how CAL programs arrange for a flexible and convenient way to present a vast amount of information [9]. This supports the fact that students are able to explore and comprehend the educational material at their own pace with the help of CAL.

Learning Tool for Students and Teachers

A successful teaching session occurs when students have successfully met the learning objectives of the lesson and are able to easily comprehend what they’ve been taught. It can be suggested that CAL has the big advantage of further supporting students to meet the learning objectives [3]. In support of this, Wang et al. conducted a study to obtain and evaluate student views on computer-simulated pharmacology experiments in Australia using a questionnaire technique. They established that 98.7% of the students successfully met their learning objectives [10]. Furthermore, it was found that student’s confidence and knowledge increased in the subject after the computer-simulated experiments. The most significant result was that all (100%) students favored virtual animal models as opposed to living animal tests in the laboratory and happily recommended learning from computer simulations to other students [10].

In addition to students, CAL benefits the teachers as well. It can be argued that CAL is able to maximize the effects of lectures and supports students by allowing them to learn in their own time [11]. This allows for better time management on the teacher’s part and a relocation of resources to other aspects of co-curricular activities. An important point to note is that, sometimes, inaccessibility to teachers and teaching material is a limiting factor for some students and the help sessions provided to students outside of teaching hours are often limited. In support of this, Hughes also believes that CAL is valuable for students when teachers are not easily accessible [11].

CAL plays a role in flipped classroom techniques as well. Learners can use resources and learn the material through CAL, and then allow for more interaction and teaching when using the learning from CAL in a future classroom session. In this way, learning is done outside the classroom through CAL prior to a classroom session, which then enhances learning.

Additional Learning and Self-Assessment

Multi-device access using cloud connectivity further enhances the applicability of CAL in terms of being able to have quick access in school, at work, or at home, maximizing productivity. Most CAL software applications include self-assessment tools—multiple-choice questions (MCQs) are an excellent way of assessing key concepts and allowing for interactive feedback on one’s performance.

(i) Study I:

Amesse et al. point out how a distinctive feature of CAL is to produce self-assessment exercises in the student’s personal time [12]. One may argue how CAL may cater better toward specific teaching arrangements that classroom lectures aren’t able to offer. For example, Baker and Dwyer claim that one teaching format that CAL does support is visual imagery learning [13]. An example of where extensive visualization and spatial learning is required is in the training of gynecology and obstetrics. As a result, it seems CAL has the power to give students that additional enhancement in their comprehension. Letterie states that the digital and imaging elements that are utilized to perform ultrasounds are tailored well for interactive CAL programs [14]. Amesse et al. studied if the extensive application of CAL was a valuable learning method for teaching third-year medical students prenatal ultrasound diagnostic skills compared with teaching using paper-based methods (PBM) [12]. A total of 36 students participated in the study over a course of six months. The course consisted of a pre-test, the CAL and PBM intervention, and a post-instruction examination. To ensure this was a fair test, the students were distributed equally per group (CAL group and PBM group). The outcome was measured by comparing the difference in score in the pre-tutorial and post-instruction examination. An important result was established about CAL after this study—students who were aided by CAL had significantly higher results in the post-tutorial exam as compared to those aided by PBM (p<0.05). Based on these results, they concluded that CAL was an effective learning method. This is one of the many studies that support the concept of CAL in developing students’ knowledge and enhancing their comprehension.

(ii) Study II:

Another study that obtained important results regarding CAL was carried out by Govindaraja et al. [15]. It came to their attention that medical students are often faced with a difficulty to conceptualize many characteristics in pharmacology. The aim was to explore if CAL was able to improve the comprehension of students and to evaluate the cognitive skills of undergraduate medical students using computer simulations employed for pharmacological practical experiments [15]. They produced a survey questionnaire for 127 students to fill based on the outcomes, advantages, and disadvantages of the CAL resource that employed simulation software. Students were required to take an MCQ test before and after the CAL period. Results were established using descriptive statistics and a proportion test. It was found that 83.3% of the students valued the computer simulations and 75% of the students stated that their comprehension had enhanced and that they had liked using CAL. Furthermore, it was revealed that almost 70% of the students had met the learning objectives, favored computer simulations over animal experiments, and were enthusiastic to recommend CAL to other students. With regards to how CAL is beneficial in pharmacology, over 80% of the students believed that complex procedures were better demonstrated using virtual animals as opposed to observing live animals. Importantly, it was concluded that CAL strengthens the understanding of class lectures, supplements the learning experience, and allows students to learn at their own pace [15].

In addition, CAL allows the students the flexibility to pause and grasp what they’ve learned and then resume, allowing them to customize according to their pace. This advantage of CAL for slow learners was perceived in the study by Govindaraja et al. and Greenhalgh [15-16]. Reviewing the literature, Kallinowski et al. examined the students' preferred method of learning between a CAL program and a lecture on teaching the management of radial fractures to 150 students in Germany [17]. The CAL group of students had video clips and exhaustive clinical information from different software to absorb, whereas the other group simply had to intake lectures. It was established that the CAL group ranked their learning experience as 15%-20% more valuable than the lecture group [17]. This shows that there is a significant difference between CAL and classroom teaching.

(iii) Study III:

Devitt and Palmer evaluated different CAL methods while teaching 90 students in Australia face to face [18]. The CAL groups were divided according to the following categories: problem-based, free-text response, and didactic. The method to measure the outcome was by analyzing the performance of written assessments. It was noted that students in the CAL didactic group scored significantly better in comparison to the other three groups [18]. Again, this was in support of CAL and how it assists students with their learning to perform better. Hilger, Hamrick, and Denny studied two groups involving a total of 77 students in the USA [19]. The first group experienced a CAL instruction program on strep pharyngitis in comparison to the second group, which received no teaching intervention. The performance on an MCQ assessment was the method operated to measure the outcome of CAL. It was found that the CAL group performed significantly higher in the assessment [19].

(iv) Study IV:

A large-scale study involving 328 students in Australia was carried out by Lyon et al. [20]. The aim was to measure the effectiveness of an interactive CAL program involving images, text, and hypertext in comparison to printed text materials, in explaining the management of anemia and chest pain. The outcome was weighed by the performance on MCQs regarding problem-solving. Although there was no difference in performance between the two groups, the CAL group took 43% less time to attain the same level of proficiency [20].

(v) Study V:

Schwartz and Griffin studied 75 students on their computer-based performance feedback in making diagnoses [21]. Schwarz and Griffin found how students using CAL with simulated patient cases followed by feedback flourished in diagnostic accuracy. One may argue that this is one of the compulsory skills required to be a proficient clinician. It seems inevitable that outstanding teaching and training will lead to invaluable performance as a doctor. Ideally, if doctors are prompt in diagnosing illnesses, patients will receive treatment quickly and, hopefully, their lives will be saved. As a result, it is advisable to implement CAL in addition to the training and teaching received through conventional academics methods.

(vi) Study VI:

Problem-solving is one of the key skills required for a physician. A study conducted by Weverling et al., consisting of 103 students in the Netherlands, assessed the significance of a CAL program with simulated patients in addition to classroom teaching in neurology in comparison to a group just receiving classroom teaching [22]. It consisted of a problem-solving assessment and a knowledge test. It was found that the CAL group scored significantly better in the assessed problem-solving task but not in the knowledge test [22]. With the support of the above literature, one can argue how CAL is definitely a fruitful practice for developing an understanding of the medical material and giving students that extra boost in their learning.

Computer Simulations

Computer simulations are very resourceful when students are able to visualize the effects of drugs and comprehend complex experiments better via simulations as opposed to practical observations in a laboratory. With the assistance of CAL, time-consuming and complex experiments can be performed [3]. Furthermore, cost and time is a big part of training students, so if these two variables are managed with the help of CAL, it would be a big advantage for health care, as the resources can be redirected to research and development.

Global Convenience and Availability

Another advantage of CAL, as Haq and Dacre [7] believe, is that “the same material is available to a large number of people over a wide geographical area, which standardizes the learning experience...” [7]. Greenhalgh agrees that CAL is convenient and flexible [16]. The literature continues to argue how some computer applications may reduce the necessity of face-to-face lectures and seminars, which, as a result, provides an arrangement for staff and students with geographical constraints [16].

Disadvantages of CAL

Regardless of the many benefits that CAL has to offer, there are a few disadvantages too. As pointed out by John et al., CAL limits direct interaction with the living tissue and the observation of variations in responses in the living tissue [3]. It may be suggested that with direct interaction, students are able to notice more visible results during an experiment, as a result, having the chance to make better conclusions. More so, using CAL means that the chance for students to experience and participate in a practical experiment becomes scarce [3]. Partaking in a practical experiment may be more memorable for students and help them retain information easily. The importance of having a teacher in the classroom setting, who can provide adequate mentorship, direct supervision, and instruction on the fly to help engage learners, cannot be underestimated. Live teachers can also engage the audience with questions that can stimulate learning as well, which is not apparent with CAL.

Technical Challenges and Access Issues

Technology comes with its own set of challenges. Students have to rely completely on electronic devices for CAL. Consequently, technical issues, which can be infrequent, may lead to disruptions in the educational process. Furthermore, the implementation of CAL is not always straightforward. The development of CAL software is labor intensive, requiring appropriate hardware, backup, and frequent upgrading [3]. It can be suggested that some teachers/lecturers may lack expertise in understanding how specific software works and, so, won’t be able to explain clearly to the students how to utilize it and maximize the benefits of that software. Some teachers have an insufficient amount of expertise in customizing or even using software applications and, as a consequence, are obliged to ask for help from information technology staff [7]. This can become quite time-consuming and, as a result, hinder the pace of learning. A study conducted by Church, Elves, Inman, and Scriven, which involved surveying 489 medical registrars, found that 76% had access to the Internet at work and 30% at home, whereas 24% reported no access to the Internet at home [23].

Computer Education and Training

As the technology expands further, it must be remembered that to gain full benefits from CAL, all students and healthcare workers should receive informative training in hardware and software use. Only then can CAL be beneficial on a large scale. With the establishment of electronic patient records, it seems crucial that all healthcare workers should have at least basic computer skills.

Leadership and Effective Integration

It will take motivated leadership to effectively integrate CAL into the curriculum. Directors of medical education in medical schools, as well as in postgraduate training programs, will need to work together to develop strategies to increase flexibility in incorporating CAL in existing and future models of medical education and physician training.

Reviewing Input and Collaboration

A review committee will need to oversee the seamless incorporation of CAL into the medical school curriculum. In addition, it will facilitate collaboration between medical education and post-graduate training programs to ensure the appropriate preparation of medical students for a successful career in a profession that is becoming increasingly dependent on automated computer systems.

## Conclusions

With the advancement of technology, healthcare education should, just as its practice is, take advantage of computer systems for the achievement of its goals. Learning from the research carried out to investigate the impact of CAL and the usefulness of incorporating CAL-based tools in conventional educational methods may improve learning through imaginative simulations, interactive collaboration, and intuitive feedback.
